# HIV Preintegration Transcription and Host Antagonism

**DOI:** 10.2174/1570162X21666230621122637

**Published:** 2023-09-05

**Authors:** Yuntao Wu

**Affiliations:** 1 Center for Infectious Disease Research, George Mason University, Manassas, Virginia, United States

**Keywords:** HIV unintegrated DNA, preintegration transcription, chromatin, histone deacetylase inhibitor, Nef, Tat, Rev, Vpr

## Abstract

Retrovirus integration is an obligatory step for the viral life cycle, but large amounts of unintegrated DNA (uDNA) accumulate during retroviral infection. For simple retroviruses, in the absence of integration, viral genomes are epigenetically silenced in host cells. For complex retroviruses such as HIV, preintegration transcription has been found to occur at low levels from a large population of uDNA even in the presence of host epigenetic silencing mechanisms. HIV preintegration transcription has been suggested to be a normal early process of HIV infection that leads to the syntheses of all three classes of viral transcripts: multiply-spliced, singly-spliced, and unspliced genomic RNA; only viral early proteins such as Nef are selectively translated at low levels in blood CD4 T cells and macrophages, the primary targets of HIV. The initiation and persistence of HIV preintegration transcription have been suggested to rely on viral accessory proteins, particularly virion Vpr and *de novo* Tat generated from uDNA; both proteins have been shown to antagonize host epigenetic silencing of uDNA. In addition, stimulation of latently infected resting T cells and macrophages with cytokines, PKC activator, or histone deacetylase inhibitors has been found to greatly upregulate preintegration transcription, leading to low-level viral production or even replication from uDNA. Functionally, Nef synthesized from preintegration transcription is biologically active in modulating host immune functions, lowering the threshold of T cell activation, and downregulating surface CD4, CXCR4/CCR5, and HMC receptors. The early Tat activity from preintegration transcription antagonizes repressive minichromatin assembled onto uDNA. The study of HIV preintegration transcription is important to understanding virus-host interaction and antagonism, viral persistence, and the mechanism of integrase drug resistance. The application of unintegrated lentiviral vectors for gene therapy also offers a safety advantage for minimizing retroviral vector-mediated insertional mutagenesis.

## 1 INTRODUCTION

Genome integration into host chromatin is an obligatory step for the replication of retroviruses [1-3]; simple retroviruses such as murine leukemia virus (MLV) encode only structural proteins and rely on cell division to gain access to the nucleus for gene expression and integration [4]. Once inside the nucleus, viral episomal genomes are quickly assembled into suppressed chromatin [5], and only subsequent integration can relieve this host restriction to allow gene expression to occur [6]. In contrast, complex retroviruses such as HIV-1 contain multiple accessory proteins, including Tat, Rev, Nef, Vif, Vpu, and Vpr, and have a great capacity to antagonize host antiviral mechanisms and to enter the nucleus without relying on cell division [7, 8].

Like other retroviruses, HIV was believed to replicate through obligatory steps of reverse transcription and subsequent integration of the viral genome; indeed, integration has been shown to be essential for HIV replication in CD4 T cells and macrophages, the primary targets of HIV infection [9-14]. Yet early mutagenesis studies of the HIV integrase protein have found that certain integrase mutants were fully competent for HIV particle production, generating wild-type levels of extracellular viral p24 antigen and Env in two HTLV-transformed T cell lines, MT-4 and Mo-T [15]; similarly, productive replication of HIV integrase mutants has also been reported in MT-4 and C8166 cells [16]. These early observations have called into question the essential role of integration in the HIV life cycle. At the same time, in HIV infection of its natural target cells, integration has been shown to be essential for productive HIV replication in primary blood CD4 T cells [13, 14, 17], transformed T cell lines [9, 11, 13], peripheral blood mononuclear cells (PBMC), and PBMC-derived macrophages [10, 12], similar to earlier observations of the requirement for viral integration in other retroviruses [1-3]. The unexpected replication of HIV integrase mutants in certain T cell lines (*e.g* MT-4 and Mo-T) [15, 16] but not others (*e.g*. CEM-50) [13] may result from possible complementation of HIV with closely related HTLV, as suggested [13, 18]; both MT-4, Mo-T, and C8166 are HTLV-transformed T cells carrying integrated copies of the HTLV provirus. Indeed, functional similarities and complementation between HIV Tat and HTLV Tax, and HIV Rev and HTLV Rex, have been previously documented [19-26]. Certainly, it has also been shown that the presence of a copy of an integrated provirus in a cell can allow viral complementation and the co-replication of multiple viruses in the cell without the need for new integration [27, 28]. In addition, rare, non-virus mediated integration or recombination between HIV and HTLV may also contribute to low-level viral gene expression or replication in the absence of HIV-mediated integration [16, 29]. However, the non-integrase-mediated integration is characterized by extremely low efficiency, deletions at the viral-cellular DNA junction, or oligomerization of viral DNA, and thus is unlikely to contribute to the replication of HIV integrase mutants in a significant manner in short-course replication assays [29].

Although not able to replicate in most CD4 T cells, a subset of integration mutants, with mutations in the catalytic residues, have been found to activate the LTR-driving reporter in a HeLa-reporter cell line, suggesting possible viral activities derived from unintegrated viruses that can stimulate cellular activities or the LTR promoter [11, 30, 31]. However, the ability of these integrase mutants to activate the LTR reporter cells could also be derived from the intrinsic leakiness of the LTR-based reporter system [32, 33] rather than being conclusive evidence of *de novo* synthesis of the HIV Tat protein in the absence of integration. It has been demonstrated that HIV Tat-independent transactivation of the LTR promoter can be achieved both by stimulation from non-viral factors, such as cytokines and mitogens present in virus preps [19, 33-40] or simply by HIV structural proteins, such as Env from infecting viral particles, as Env has been shown to stimulate the LTR promoter [41]. Because of these mechanistic uncertainties, the notion that HIV may express genes or even replicate in the absence of integration was met with skepticism for years.

## 2 PREINTEGRATION TRANSCRIPTION IS A NORMAL EARLY PROCESS OF HIV INFECTION THAT LEADS TO THE SYNTHESIS OF THE NEF PROTEIN

Nef is the first HIV accessory protein that has been demonstrated by western blots to be translated prior to integration in HIV infection of its natural target cells, including blood resting and activated CD4 T cells [13, 14] and PBMC-derived macrophages [12]. In HIV-1 latent infection of blood resting CD4 T cells, the Nef protein was the only newly synthesized viral protein detectable at a low level by western blot when integration was not detected and productive HIV replication had not occurred [14]. To confirm the early synthesis of the Nef protein from the unintegrated genome of wild-type (Wt) HIV, an integrase mutant, HIV-1_IN/D116N_ (D116N) was also used and was found to produce Nef [14]; the protein level of Nef was also found to be comparable at an early time between Wt and D116N infection, demonstrating the capacity of HIV to express the *nef* gene early without integration in blood T cells [14]. In contrast to the limited transcription of *nef* in resting T cells, in HIV-1 infection of CD3/CD28-activated or transformed T cells, HIV-1 preintegration transcription was found to be more robust and capable of transcribing all three classes of viral transcripts, namely multiply-spliced, singly-spliced, and unspliced [13]. Yet, as in resting T cells, only the *nef* gene was measurably translated. Poon *et al.* have also found that the synthesis of Nef is dependent on HIV virion Vpr in transformed SUP-T1 T lymphoblast cells; Vpr was found to greatly enhance expression from uDNA [42, 43].

A kinetic analysis revealed that preintegration transcription was transient in proliferating T cells; the viral transcripts were detectable as early as 6 hours, peaked at 12-24 hours, and then diminished afterwards [13]. Levels of preintegration transcription during the early time period (6-12 hours) were similar between Wt and D116N infection. The early *nef* transcripts from both Wt and D116N infection were also found to be completely resistant to the diketo acid integrase inhibitor L-708,906. Thus, the study concluded that preintegration transcription is a normal early process occurring in productive HIV infection, and did not result from possible peculiarities of certain integrase mutants [13]; unintegrated Wt HIV DNA has the full capacity to synthesize all classes of viral transcripts, both the early multiply-spliced, and the late, singly-spliced and unspliced, prior to integration [13].

**Fig. (1):**
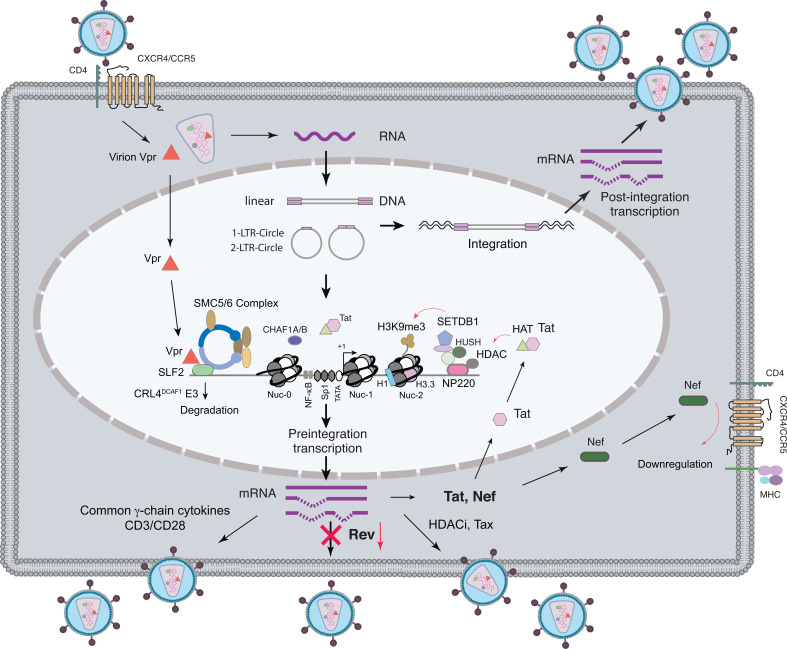
HIV infection and preintegration transcription. The process of HIV infection leading to preintegration transcription is illustrated here. Following viral entry and reverse transcription, three forms of uDNA are formed, the linear, 1-LTR-circles, and 2-LTR-circles. The linear DNA is believed to be the precursor for integration which is necessary for productive HIV infection. In the absence of integration, HIV uDNA forms minichromatin and is epigenetically suppressed by host proteins such as the SMAC5/6 complex, which is recruited to uDNA by SLF2. Other host proteins such as the histone chaperones CHAF1A/B are also involved in silencing HIV uDNA. In addition, the histone methyltransferase SETDB1 and the HUSH complex may be recruited to HIV uDNA by host proteins similar to NP220. Epigenetically suppressed uDNA chromatin is characterized with histone modifications such as low levels of H3 and H4 acetylation, H3K4 dimethylation, and high levels of H3K9 trimethylation, and the presence of histone deacetylases (HDAC), histone variant H3.3, the H1 linker histones, and nucleosomes that cover the DNase hypersensitive site (NucDHS). HIV virion Vpr antagonizes host epigenetic silencing by binding to SLF2, causing its degradation *via* CRL4^DCAF1^. Vpr-mediated removal of the SMAC5/6 complex increases chromatin accessibility of uDNA to initiate low-levels of preintegration transcription, which generates all 3 classes of viral transcripts. Only early gene products such as Nef, Tat, and Rev are translated. Tat synthesized from preintegration transcription may also antagonize uDNA silencing by mechanisms such as interaction with histone acetyltransferases (HAT). Nef synthesized from preintegration transcription can modulate host immune responses by promoting T cell activation, and downregulating surface CD4, CXCR4/CCR5, and MHC molecules. Rev is synthesized at a reduced level that may not be sufficient to mediate the synthesis of viral structural proteins. Stimulation of preintegration transcription with common γ-chain cytokines, HDACi, or HTLV-Tax can greatly upregulate preintegration transcription, leading to viral replication from uDNA.

Semi-quantitative analyses of viral transcription in the presence of integrase inhibitor suggested that HIV transcription can be divided into two phases, early transcription (preintegration transcription) and late transcription (postintegration transcription) (Fig. **1**) [13]. These two sequential phases of transcriptional activity can be distinguished in several aspects. Firstly, preintegration transcription is resistant to the integrase inhibitor L-708,906, but postintegration transcription is highly sensitive [13]. Secondly, in proliferating T cells, preintegration transcription is transient and diminishes with cell division, whereas postintegration transcription can persist following integration. Thirdly, preintegration transcription is selective and skewed towards greater levels of *nef* and *tat* transcripts and decreased levels of the *rev* transcript [12, 13]. Fourthly, in preintegration transcription, while all transcripts are transcribed, only the multiply spliced early genes, such as *nef* are selectively translated [12, 13]. This selective translation of only the multiply spliced early genes has been suggested to be partially attributable to the lack of Rev function, as overexpression of Rev can lead to low-level translation of the unspliced *gag-pol* transcript [13].

This biphasic pattern of HIV transcription is reminiscent of gene transcription from DNA viruses, in which gene transcription is divided into early and late phases based on viral DNA replication [44]; early transcription occurs before viral DNA replication, whereas late transcription occurs after DNA replication. Early transcription is mainly to synthesize viral transcriptional regulators, cellular modulators, and replicases, whereas late transcription is mostly for structural protein syntheses for particle assembly. This sequential arrangement is an efficient way to coordinate viral genome replication and particle production, and this coordination ensures the availability of viral genomes for packaging before structural proteins are synthesized. For DNA viruses, the control for this early-to-late gene expression switch is viral DNA replication, whereas for HIV, this control is likely HIV DNA integration; in proliferating T cells, only the integrated viral genome is stable for a consistent supply of viral genomes for packaging.

In proliferating T cells, the transient nature of preintegration transcription from uDNA may result from cell division that can lead to the diminishment of uDNA template. Indeed, in non-proliferating, terminally differentiated macrophages, uDNA was found to be stable, persisting in cells for at least 30 days; preintegration transcription was also found to persist for at least 30 days [12]. Additionally, preintegration transcription in macrophages is also selective and skewed towards viral early genes such as *nef* and *tat* with highly diminished *rev*, and there was no detectable viral structural protein synthesis. Only the Nef protein was found to be measurably synthesized and to persist in cells [12]. The restriction on structural protein synthesis may also result from the lack of Rev function, as the Rev transcript was at a very low level over the course of D116N infection of macrophages [12, 45].

Functionally, Nef synthesized from preintegration transcription has been shown to be biologically active. In HIV infection of resting CD4 T cells, Nef synthesized prior to integration has been shown to lower the threshold of T cell activation, facilitating productive viral replication upon T cell activation [14]. In HIV infection of proliferating T cells, Nef synthesized from an integrase mutant or in the presence of the integrase inhibitor L-731,988 effectively downregulated surface CD4 receptor on primary CD4^+^ T lymphocytes [42, 46, 47]. It has also been shown that Nef expressed from unintegrated DNA can effectively downregulate surface CXCR4 and CCR5 on an HIV Rev-dependent GFP indicator T cell, Rev-CEM [47-49]. Using Rev-CEM and pre-activated primary CD4 T cells, it was further demonstrated that Nef expressed from uDNA down-modulates cell surface major histocompatibility complex class I (MHC-I), both HLA-ABC and HLA-A31 [50]. It was suggested that HIV-mediated MHC-I downregulation and evasion of host immune responsiveness can be attributable, in part at least, to the transcription of Nef from uDNA [50].

## 3 PREINTEGRATION TRANSCRIPTION LEADS TO EARLY TAT AND REV ACTIVITIES

In HIV infection, early *tat* and *rev* transcripts are detectable in primary resting CD4 T cells, preactivated and transformed T cells, and macrophages in the absence of viral integration [12-14, 51]. However, not all transcripts are translated, though [14], and neither Tat nor Rev protein synthesis from preintegration transcription has been demonstrated. In fact, the lack of structural protein synthesis has been attributed to the lack of Rev function from preintegration transcription [13]. Demonstration of the synthesis of Tat or Rev is technically difficult using conventional tools such as western blot. However, Tat function can be quantified using LTR-driving reporters, as Tat can transactivate the LTR promoter. Using LTR reporter cell lines, it has been shown that certain integrase mutants can mediate low-level reporter expression in LTR-driving reporter cell lines [11, 30, 31, 45]. But, as described above, the LTR reporters are intrinsically leaky and can be transactivated by many factors independent of Tat. To confirm that there were indeed genuine *de novo* Tat activities produced from preintegration transcription, Aguilar-Cordova *et al*. pretreated the LTR reporter cell, 1G5 [34], with reverse transcriptase inhibitor etravirine, and then infected cells with the D116N integrase mutant and found that D116N-mediated transactivation of the LTR-reporter was greatly inhibited, demonstrating that D116N-mediated transactivation is dependent on newly synthesized HIV DNA [45]. To further confirm *de novo* Tat functions, Meltzer *et al.* also constructed two unintegrated GFP HIV reporter viruses with or without the *tat* gene. When cells were similarly infected (using equal p24 of the Tat+ and Tat− viruses), the deletion of Tat greatly diminished GFP expression (96% reduction) from uDNA. This result directly demonstrated that *de novo* Tat activities are produced from uDNA. Importantly, this uDNA-derived Tat (uTat) can functionally transactivate the unintegrated LTR promoter and antagonize host-mediated chromatin silencing of unintegrated HIV genome [45]. Nevertheless, without integration, Tat cannot stimulate LTR to the high levels seen in integrated provirus, suggesting that the sites of LTR integration and the local cellular chromatin environment may have a profound impact on LTR responsiveness to Tat transactivation [45]. It has been shown that the Tat-associated histone acetyltransferases p300 and P/CAF are preferentially important for the transactivation of integrated but not unintegrated HIV-1 LTR [52].

To demonstrate *de novo* production of Rev activity, Wu *et al.* constructed an HIV Rev-dependent indicator CD4 T cell line, Rev-CEM (available from NIH AIDS Reagent program, Cat# ARP-11467), which strictly requires Rev interaction with RRE (Rev responsive element) to turn on reporter expression [48, 49]. In contrast to the leakiness of the LTR-based reporter cells, Rev-CEM has been shown to be highly specific, only expressing reporter genes in the presence of HIV and Rev, but not in response to stimulation with cytokines, mitogens, or a Rev-negative HIV mutant [47, 49, 50, 53-57]. Using Rev-CEM, Iyer *et al.* demonstrated and quantified the *de novo* generation of Rev activities in the Rev-dependent GFP reporter cells that were infected with D116N or with Wt in the presence of the integrase inhibitor 118-D24-24 [53, 58]. Similar results of Rev-dependent GFP expression from unintegrated viruses were also reported by Sloan *et al.* [47, 50]. These studies demonstrated the generation of Rev activities from preintegration transcription. Nevertheless, without integration, the levels of GFP reporter expression were at a much lower level, although a comparable population of viral genomes was transcribing with or without integration [45, 53].

## 4 TEMPLATES FOR PREINTEGRATION TRANSCRIPTION: A LARGE POPULATION OF uDNA IS TRANSCRIPTIONALLY ACTIVE AT LOW LEVELS

The natural course of HIV infection leads to the accumulation of large amounts of uDNA in infected T cells, lymphoid tissues, and the brain [59-64]. During the asymptomatic phase of HIV infection, the most prevalent form (99%) of viral DNA in patients’ blood CD4^+^ T cells is the full-length, linear, unintegrated DNA [61, 64]. In AIDS patients with dementia, there is a 10 fold higher level of unintegrated HIV DNA than integrated DNA in the brain [62]. In cell culture conditions, a majority of reverse-transcribed HIV DNA molecules also fail to integrate [12, 13, 61, 65]. Quantification of HIV integration has demonstrated that approximately 5 to 10% of total reverse-transcribed DNA normally integrates [66-68].

Three species of uDNA exist in infected cells: the full-length linear DNA, the 1-LTR circles, and the 2-LTR circles (Fig. **1**) [18]. The circular forms were first identified in infection by other retroviruses such as avian sarcoma virus (ASV) [69, 70] and Moloney murine leukemia virus (MLV) [71]. The DNA circles consist of both 1-LTR circles and 2-LTR circles [72, 73] and constitute approximately 20-25% of total viral DNA within 24-48 hours in ASV infection [74]. In addition, the 1-LTR circles were found to be present in great excess over the 2-LTR circles in infected cells [72]. The circular DNA was suggested to localize exclusively in the nucleus, and is thus frequently used as a conventional marker of HIV nuclear migration [74, 75]. The linear DNA has been suggested to be the precursor for retrovirus integration, and circular DNA is the byproduct of failed integration [76-78]. Indeed, it has been shown that HIV circular DNA is associated with discrete nuclear complexes rather than the viral preintegration complex [79]. The 2-LTR circles can be generated by simple ligation of the linear DNA [80-83], whereas the 1-LTR circles may be generated by homologous recombination between the LTRs on the linear DNA [72, 81, 82] or from the process of reverse transcription [84-86]. The circular DNA has been suggested to be labile in proliferating T cells and may serve as a marker of active viral replication in HIV-1-infected patients [87-89]. On the other hand, recent studies have demonstrated that the 2-LTR circles are highly stable and decrease in concentration only as a function of dilution resulting from cell division [90, 91]. In non-dividing macrophages, HIV circular DNA has been found to persist for at least 30 days [12, 92].

The templates for preintegration transcription have not been clearly identified. In an early transfection experiment, all synthetic LTR-containing DNA circles were found to be active when transiently transfected into HeLa cells [93]. Nevertheless, the transcriptional activity of 2-LTR circles was an order of magnitude lower than the transfected proviral DNA carrying flanking cellular sequences. 2-LTR circles were found to be greatly increased in cells infected with integrase mutants [12, 13, 30, 31] and were thought to be the template for uDNA transcription [30, 31, 94]. Howver, surprisingly, when Iyer *et al.* conducted a flow cytometry sorting of Rev-dependent GFP reporter cells infected with D116N, they discovered that the 2-LTR circles were not found exclusively in the GFP+ cells, but were randomly distributed between GFP+ and GFP- cells [53]. In addition, in Wt HIV and D116N infection of CEM-SS T cells, levels of early preintegration transcription were comparable between Wt and D116N infection, while the 2-LTR circles were two orders of magnitude more numerous in D116N infection [13]. These studies suggest that transcription from uDNA correlates with total viral DNA rather than only 2-LTR circles [13, 53]. 2-LTR circles are minor fractions of viral DNA constituting about 0.03% of total unintegrated viral DNA when preintegration transcription was measured at 12 hours post infection in CEM-50 T cells [13]. In non-dividing macrophages, 2-LTR circles were found to accumulate 11.8% of total HIV DNA in Wt-infected cells and 37.4% in D116N-infected cells at 30 days post infection [12]. It is likely that the transcribing uDNA templates are from predominant DNA species such as the full-length linear DNA and/or the 1-LTR circles, rather than only the 2-LTR circles [53].

Iyer *et al.* further quantified HIV preintegration transcription of Rev using the Rev-dependent GFP indicator cells, and found that preintegration transcription occurs on a much larger scale than previously thought [53]; the transcribing uDNA population was comparable to that derived from integrated provirus in a single-cycle HIV infection (GFP+ cell population: D116N, 9.6%; Wt, 14.6%). At the same time, the uDNA templates transcribed universally at a much lower level than proviral DNA (GFP intensity: D116N, 52.29; Wt, 468.99) [53]. Similar results were also observed in env-pseudotyped, single-cycle HIV infection of IL-4-treated resting CD4 T cells [95]; while the percentages of GPF+ cells (measurement of Nef) were comparable with or without integration (12% *versus* 7.2%), only low-expression GFP+ dim cells but not high-expression GFP+ bright cells were generated when integration was inhibited (GPF+ bright cells: 7.4% with integration *versus* 0.2% without integration). These results suggest a clear distinction between episomal uDNA and proviral template in transcribing genes from the LTR, and in responsiveness to Tat and Rev stimulation [45, 52]. Even so, given the large population of transcribing uDNA, an unintegrated lentiviral vector can be as effective as an integrated vector for expressing genes in non-dividing cells with the use of proper internal promoters [96-98].

## 5 HOST ANTAGONISM IN SILENCING PREINTEGRATION TRANSCRIPTION

The accumulation of large amounts of uDNA in cells and the body may trigger host antagonism in silencing preintegration transcription [6]. It has been suggested that similar to integrated provirus, unintegrated HIV can establish a durable latency in non-dividing resting CD4 T cells that can be re-activated by the PKC activator prostratin and the histone deacetylase inhibitor trichostatin A, suggesting the assembly of repressed chromatin structures in episomal HIV genomes [95, 99]. In HIV infection of macrophages, it has also been suggested that repressed minichromatin is assembled onto uDNA [45, 100] that can be induced to express genes with histone deacetylase inhibitors (HDACi) [100].

Details of host silencing of retrovirus uDNA have begun to emerge with the studies of minichromatin modification. Nucleosomes were found to be quickly assembled onto the HIV-1 uDNA LTR within 4 hours post-infection [45, 101] and exhibited a profile typical of silent chromatin, including low levels of H3 and H4 acetylation and H3-K4 dimethylation, high levels of H3-K9 trimethylation [100], and the assembly of a nucleosome that covers the DNase hypersensitive site (NucDHS) [101]. In addition, relatively higher levels of repressive histone deacetylase 1 (HDAC-1) were found to be associated with unintegrated minichromatin than with integrated proviral chromatin in the absence of Tat [45]. Recently, Geis *et al.* also demonstrated that core histones, the histone variant H3.3, and H1 linker histones are all deposited onto extrachromosomal HIV-1 uDNA early after infection with a Vpr-negative virus [102]; in the absence of Vpr, HIV uDNA is assembled into minichromatin characterized by posttranslational histone modification of transcriptionally inactive genes, including high levels of H3K9 trimethylation and low levels of H3 acetylation [102]. In addition, Geis *et al.* have further demonstrated that in the absence of Vpr, there is a high degree of silencing of uDNA in certain T cell lines, such as K562 and Jurkat [103]. In particular, in K562 cells, uDNA is loaded with histones of high silencing marker H3K9 trimethylation and low active marker H3 acetylation. In addition, uDNA also carries low H3K27 trimethylation, similar to low H3K27 trimethylation levels of silent host globin genes.

Virion Vpr has been shown to greatly antagonize the host silencing machinery, restoring gene expression from uDNA [42, 43, 103]. The capacity of Vpr to antagonize host silencing of uDNA likely results from its capacity to target multiple chromatin-modifying proteins for degradation. Vpr has been shown to induce the degradation of HDAC1 and HDAC3 on HIV-1 LTR in latently infected J-Lat cells, and as a result, makers of active transcription were recruited to the viral promoter for activation [104]. In addition, Vpr and Vpx have been found to complex with the human silencing hub (HUSH) complex to cause HUSH degradation [105]. Vpr has also been found to associate with the Cul4A-DDB1^DCAF1^ ubiquitin ligase to deplete CTIP2, removing it from the complex of heterochromatin-promoting enzymes for silencing HIV LTR [106]. Vpr can also use CRL4^DCAF1^ E3 ligase to degrade histone deacetylase SIRT7 [107]. Recently, using CRISPR-Cas9 knockout screening, Dupont *et al.* identified SMC5-SMC6 complex localization factor 2 (SLF2) as the Vpr target responsible for silencing unintegrated HIV-1; SFL2-dependent recruitment of SMC5/6 complex to HIV uDNA leads to compaction of viral chromatin and loss of active histone marks. Vpr degrades SLF2 *via* CRL4^DCAF1^, thereby increasing chromatin accessibility of uDNA for activating gene expression [108]. In addition to SLF2, two histone chaperones CHAF1A and CHAF1B have also been recently identified as essential factors for silencing HIV uDNA in the absence of Vpr [109]. However, the phenotypes of CHAF1A/B on silencing HIV uDNA do not appear to be affected by Vpr [109].

In addition to Vpr, HIV Tat has also been suggested to partially antagonize host chromatin silencing [45]; in the absence of Tat, uDNA minichromatin is gradually silenced but remains highly inducible by HDACi. It has been suggested that Tat functionally antagonizes uDNA minichromatin repression to maintain persistent expression of viral early genes such as Nef in macrophages for at least 30 days [12]. Tat-mediated viral persistence may establish a viral reservoir in macrophages where uDNA can persist for a long period of time [45]. Tat antagonizes uDNA chromatin silencing likely through its ability to interact with histone acetyltransferases (HAT) such as p300/CBP and P/CAF [52, 110, 111], Tip60 [112], and hGCN5 [113]. Consistent with the stimulatory role of Tat, HTLV-1 Tax has also been shown to interact with HAT [114] and acts similarly to HIV-1 Tat to stimulate HIV uDNA transcription and the replication of HIV integrase mutant in Tax overexpression cells [25, 26, 45].

Similar chromatin silencing of viral uDNA has also been observed in infection by other retroviruses such as MLV [115]. In MLV infection, core histones are rapidly loaded onto unintegrated virus DNAs following viral nuclear entry but prior to integration [5]. The host factors responsible for silencing MLV uDNA have been identified using CRISPR-Cas9 knockout screening; it was shown that the DNA-binding protein NP220, the HUSH complex (MPP8, TASOR and PPHLN1), the histone methyltransferase SETDB1, and other host factors are required for uDNA silencing [116]. Mechanistically, NP220 recruits the HUSH complex, SETDB1, and the histone deacetylases HDAC1 and HDAC4 to silence MLV uDNA [116]

## 6 HIV PRODUCTION AND REPLICATION FROM uDNA FOLLOWING STIMULATION WITH HISTONE DEACETYLASE INHIBITORS OR COMMON GAMMA-CHAIN CYTOKINES

Although certain HIV integrase mutants have been shown to replicate in HTLV-transformed T cell lines [15, 16], such mutants did not replicate in primary resting CD4 T cells, pre-activated and transformed T cells, or macrophages in the absence of HTLV complementation [10-14]. At the same time, Cara *et al.* [17] and Kantor *et al.* [100] reported low-level p24 production of an unintegrated HIV-1 mutant in PBMC-derived macrophages, either unstimulated [17] or stimulated with HDACi in the form of various short-chain fatty acids (SCFAs) [100]; it was suggested that unintegrated HIV-1 genomes are organized into chromatin structures enriched with histone modifications typical of transcriptionally silenced chromatin. Stimulation of cells with HDACi notably increases gene expression from unintegrated HIV-1 [45, 100], leading to a low-level (2-3-fold) increase in the production of HIV p24 [17, 100].

HDACi-independent replication of unintegrated HIV has also been demonstrated in the infection of blood resting CD4 T cells stimulated with common gamma-chain cytokines [95], a condition mimicking HIV infection and replication in lymphoid tissues where cytokines are found to be highly enriched [117-119]. Although HIV preintegration transcription is limited to early genes in resting CD4 T cells [14], culturing resting T cells with common gamma-chain cytokines, including IL-2, IL-,4, IL-7 and IL-15 relieved this restriction and permitted HIV-1 replication in the absence of integration [95]; in these cytokine-treated and post infection-activated T cells, mutating the HIV integrase active sites (D116N, D64E) or using an integrase inhibitor, raltegravir, did not block HIV-1 replication, although both effectively blocked viral integration. Importantly, viruses released in the presence of raltegravir were shown to be infectious and replication-competent in new target cells [95]. Remarkably, Vpr was found to be absolutely required for HIV-1 gene expression and virus replication from uDNA in cytokine-treated T cells [95]; Vpr is only stimulatory for provirus replication in these conditions [95]. It has been suggested that both virion-associated Vpr and newly synthesized Vpr are critical for uDNA expression [42, 43, 95] and virus replication, respectively [95].

Given the *in vitro* integration-independent HIV replication observed [95], a critical question is whether such replication can occur *in vivo*. Certainly, integrase inhibitors are used in drug combinations to treat HIV infection and have shown effectiveness in bringing about faster plasma viral load decay when used in combination with reverse transcriptase (RT) inhibitors [120-122]. However, in the animal monotherapy trial of the integrase inhibitor L870812, the drug only inhibited acute SIV replication in rhesus macaques (treatment from day 10 after infection), and failed to control viremia when used at a later time (day 87 post infection) [123]; five of the six animals had high viral loads that could not be suppressed by the drug [123]. Importantly, the integrase drug resistance was not attributed to mutations in the integrase gene, as the recovered viruses were found to carry wild-type integrase [123]. As such, the mechanism of drug resistance to L870812 is not understood, but could be related to dysregulation of the immune system at late disease stages, when inflammatory cytokines and other immune stimulators accumulate [95, 99, 100]. These conditions may greatly upregulate transcription from uDNA, as is seen in cell culture conditions [95, 100].

Somewhat consistent with the animal trial [123], in human HAART intensification studies, the integrase inhibitor raltegravir reduced neither residual viremia nor the total amount of viral DNA, although it blocked viral integration and increased the amounts of unintegrated 2-LTR circles [124-126]. Intriguingly, multiple clinical studies have found that the integrase inhibitor-resistant viruses from at least half of the patients do not have mutations in the integrase region [127-129]. The underlying mechanisms are largely obscure. It has been suggested that mutations in the HIV Env gene region may alter the HIV entry pathway and promote cell-cell transmission that may evade integrase inhibitors [130]. It remains to be determined whether HIV cell-cell transmission contributes to HIV integrase drug resistance *in vivo*. It also remains to be determined whether uDNA could contribute to residual viremia and some degree of HIV replication *in vivo,* either directly or through complementation with integrated proviral reservoirs. Recent mathematical modeling has suggested that uDNA could contribute to 20% of total viremia in HIV infection [131].

## CONCLUSION

The natural course of HIV infection leads to the accumulation of large amounts of unintegrated HIV DNA [59-63], a likely reflection of the fact that integration is frequently inefficient. In the absence of integration, retroviral uDNA is rapidly chromatinized and silenced in the nucleus by host factors [6]. In spite of this potent host epigenetic silencing, HIV has evolved the capacity to initiate low-level transcription from uDNA [12-14, 95]. This capacity is largely attributed to viral accessory proteins, particularly Vpr and Tat, that can functionally antagonize host epigenetic silencing (Fig. **1**) [42, 43, 45, 108]. Given that post-integration transcription is the persistent and dominant transcriptional activity, the question is why pre-integration transcription occurs and is important for HIV. The answer likely lies in the target cells. Simple retroviruses rely on cellular mitotic activity to gain access to the nucleus. As such, uDNA is rapidly diminished in dividing cells, and the syntheses of structural proteins in the absence of integration do not appear to be advantageous. Complex retroviruses such as HIV can gain access to the nucleus of non-dividing cells such as resting CD4 T cells and macrophages. In these primary targets of HIV, even when HIV uDNA is restricted for integration [14, 132], it can still persist for weeks to months and be active for low-level transcription of regulatory genes such as *nef* and *tat* to modulate host cells [12, 14, 95]. It has also been suggested that a transcribing uDNA may be more stable [6]. HIV may have also evolved a strategy of multiple infection and viral complementation to gradually break down cellular barriers [27, 28]. uDNA selectively expresses two early regulatory proteins, Nef and Tat [12-14, 45]. Both are known to modulate T cell activity [14, 133, 134], promoting T cell activation to increase the chances of productive infection during multiple infections [27]. uDNA can also transcribe genomic RNAs [12-14], which are rescuable for co-packaging by integrated provirus for continuous viral replication [27]. This complementation between integrated provirus and unintegrated HIV in a cell would enable the survival of large numbers of unintegrated genomes and avoid the loss of viral genetic diversity [27, 28, 135].

For host cells, silencing uDNA in the nucleus may be an innate response for maintaining the transcriptional fidelity of the host. In addition, silencing uDNA may also avoid viral evasion of host immune surveillance, as Nef expressed from uDNA has been shown to downregulate HMC [50]. The molecular details of host silencing preintegration transcription have begun to emerge with the recent identification of host factors [6, 108, 116], but many critical questions remain to be answered. For example, it is unknown how epigenetic silencing or activation of uDNA affects the ability of the viral genome to integrate. A possible direct linkage between preintegration transcription and postintegration transcription in the retroviral life cycle is also missing.

The *in vivo* role of preintegration transcription in viral pathogenesis and persistence needs to be examined. *In vitro*, preintegration transcription from uDNA can be greatly upregulated by cytokines or histone deacetylase inhibitors, leading to low-level production of replication-competent viruses [95, 99, 100]. These studies suggest that viral uDNA genomes may serve as an inducible alternative reservoir in addition to integrated provirus [136]. In treating HIV infection, integrase inhibitors are used in drug combinations. However, the drugs do not appear to be effective for reducing residual viral reservoirs. Many of the drug-resistant viruses recovered from patients do not carry mutations in the integrase gene. The underlying mechanisms of drug resistance are largely obscure. A potential role of preintegration transcription in HIV integrase drug resistance also awaits investigation.

Basic research on HIV preintegration transcription has also stimulated the use of unintegrated lentiviral vectors for gene therapy [96-98, 137-141] and vaccines [142]. Unintegrated vectors offer a safety advantage as they do not disrupt cellular genes [137, 138]. These unintegrated vectors have demonstrated high efficiency in mediating stable gene expression in terminally differentiated cells [96-98]. For example, gene expression from an unintegrated lentiviral vector in mouse liver was found to be potent and sustained, lasting for 6 months [143]. Recently, an HIV-based unintegrated vector has also been shown to be efficient and sustained in rodent ocular and brain tissues and has corrected retinal disease in a mouse model [98].

## LIST OF ABBREVIATIONS

GFP: Green Fluorescent Protein
HIV: Human Immunodeficiency Virus
HTLV: Human T-lymphotropic Virus
LTR: Long Terminal Repeats
MHC: Major Histocompatibility Complex
Nef: Negative Factor
Rev: Regulator of Expression of Virion Proteins
Tat: Trans-Activator of Transcription
Vpr: Viral Protein R
